# MMP-9, Vertebrobasilar Ectasia and Vertebral Artery Dominance in Vertigo or Dizziness Patients With Vascular Risk Factors

**DOI:** 10.3389/fneur.2020.00931

**Published:** 2020-08-25

**Authors:** Huai Liang Zhang, Yan Fang Peng, Dao Pei Zhang, Dan Li, Fei Xiang Liu, Min Zhao, Suo Yin, Jia Xu Liang, Tian Tian Wei

**Affiliations:** ^1^Department of Neurology, The First Affiliated Hospital of Henan University of Chinese Medicine, Zhengzhou, China; ^2^Department of Cerebrovascular Disease, The Fifth Affiliated Hospital of Sun Yat-sen University, Zhuhai, China; ^3^Department of Image, People's Hospital Affiliated to Henan University of Chinese Medicine, Zhengzhou, China; ^4^Clinical Medical Testing Center, People's Hospital Affiliated to Henan University of Chinese Medicine, Zhengzhou, China

**Keywords:** matrix metalloproteinases, vertebrobasilar ectasia, vertebral artery dominance, vertigo, stroke

## Abstract

**Background and Purpose:** Although vertebrobasilar ectasia (VBE) is diagnosed with increasing frequency, it is not clear whether this is because of altered hemodynamics caused by the effects of matrix metalloproteinases (MMPs) and/or vertebral artery dominance (VAD). Therefore, we investigate the relationship between plasma levels of MMPs and VBE in patients with vertigo or dizziness who also have vascular risk factors, in order to determine whether high levels of MMPs in VBE are independent of VAD.

**Methods:** We prospectively studied 285 patients with vertigo or dizziness and at least one vascular risk factor. Plasma levels of MMPs, tissue inhibitor of metalloproteinases (TIMPs) and cathepsin L were measured. Subjects were classified as VBE-negative or VBE-positive, who were further classified based on the presence of VAD with magnetic resonance angiography. Acute ischemic stroke was screened by diffusion-weighted imaging, generally after bedside evaluation and the drawing of blood samples. Receiver operating characteristic (ROC) curves were applied to evaluate the utility of these potential biomarkers in predicting risk for ischemic stroke.

**Results:** The prevalence of VBE in patients with vertigo or dizziness was 16.5%. Of the 82 patients with ischemic stroke, 14 strokes involved the cortex or subcortex. MMP-9 levels were significantly higher in the VBE-positive group than in the VBE-negative group (*P* = 0.022). There was a significant difference in the risk of posterior circulation ischemic stroke between the VBE-positive group and the VBE-negative group (*P* = 0.002). Levels of MMP-2 and cathepsin L tended to be higher in the VBE-negative group (*P* = 0.054, *P* = 0.060, respectively). Compared with the non-VAD subgroup, levels of MMP-2,−3,−9, TIMP-1,−2, and cathepsin L were similar in the VAD subgroup. ROC analysis showed that MMP-9 predicted risk for ischemic stroke (AUC = 0.582, 95%CI, 0.510–0.654, *P* = 0.030).

**Conclusions:** MMP-9 was associated with VBE and independent of VAD. High levels of MMP-9 may predict risk for ischemic stroke in patients with vertigo or dizziness who also have vascular risk factors.

## Introduction

Vertebrobasilar ectasia (VBE) is defined as1 basilar artery (BA) >4.5 mm (1) and/or vertebral artery >4.0 mm (2). The condition affects 0.06–6% of healthy individuals and 1–11% of stroke patients (3). VBE is a main subtype of vertebrobasilar dolichoectasia (VBD) and a predictor of further ischemic stroke or hemorrhage (4, 5), as well as increased risk for death (3). The essential pathological changes of VBE involve extracellular matrix or smooth muscle cells of the tunica media and attenuation or fracture of the internal elastic lamina (6, 7). No specific therapy has been established for the treatment of patients with dolichoectasia. Surgical interventions have largely aimed to relieve compressive symptoms (7, 8). Elucidating the pathophysiological mechanisms of VBE may allow for more effective treatment.

An imbalance in matrix metalloproteinases (MMPs)/tissue inhibitors of metalloproteinases (TIMPs) and the altered hemodynamics resulting from unequal vertebral artery flow are thought to contribute to VBE by inducing vascular remodeling in arterial walls (1–3). On the one hand, MMPs are enzymes that degrade components of the extracellular matrix. TIMPs are endogenous inhibitors of MMPs; the balance between MMPs/TIMPs regulates extracellular matrix turnover, including effects mediated by cathepsin as well as remodeling during normal development and pathogenesis (9). A study focused on MMPs and arterial remodeling in the brain among individuals with and without HIV infection revealed that dolichoectasia was associated with high MMP-9 expression alone, high MMP-9 expression combined with low TIMP-2 expression, or high MMP-9 expression combined with expression of caspase 3 (10). A prospective study demonstrated that MMP-2 played a role in the etiology of dolichoectasia of cervical and intracranial carotid arteries (11). A mouse model study showed that dolichoectasia was associated with high levels of MMP-2 and MMP-9 (12). A clinical study demonstrated that dolichoectasia was associated with lower plasma levels of MMP-3 (13). On the other hand, one important study demonstrated that vertebral artery dominance (VAD) contributed to basilar artery curvature and peri-vertebrobasilar junctional infarcts (14). Another study showed that the incidence of posterior circulation infarction (PCI) was significantly higher in patients with BA curvature than in patients with straight basilar arteries (BAs). The incidence of BA curvature is increased in VAD patients, and the incidence of PCI is increased in VAD patients, especially in patients with posterior inferior cerebellar artery infarction or BA infarction (15). A longitudinal study of geometric changes to the BA included 154 subjects with normal vertebrobasilar arterial systems. After the results of magnetic resonance angiography were evaluated, patients were assigned to one of two groups: non-VAD and VAD. The study's results confirmed that, while bending of the BA depends on dominance of the VA, increased BA length is related to advanced age (16). Both of the mechanisms described above may contribute to VBE. Additional research will be necessary to determine whether VAD and MMP-2/3/9 are associated with or independent of VBE.

In this study, we evaluated patients with dizziness or vertigo and at least one vascular risk factor who were at an increased risk for posterior circulation ischemic events (17, 18) in order to test the hypothesis that MMP-2/3/9 are associated with VBE and to determine whether high levels of MMP-2/3/9 in VBE are independent of VAD.

## Materials and Methods

### Study Population

**Figure 1:**
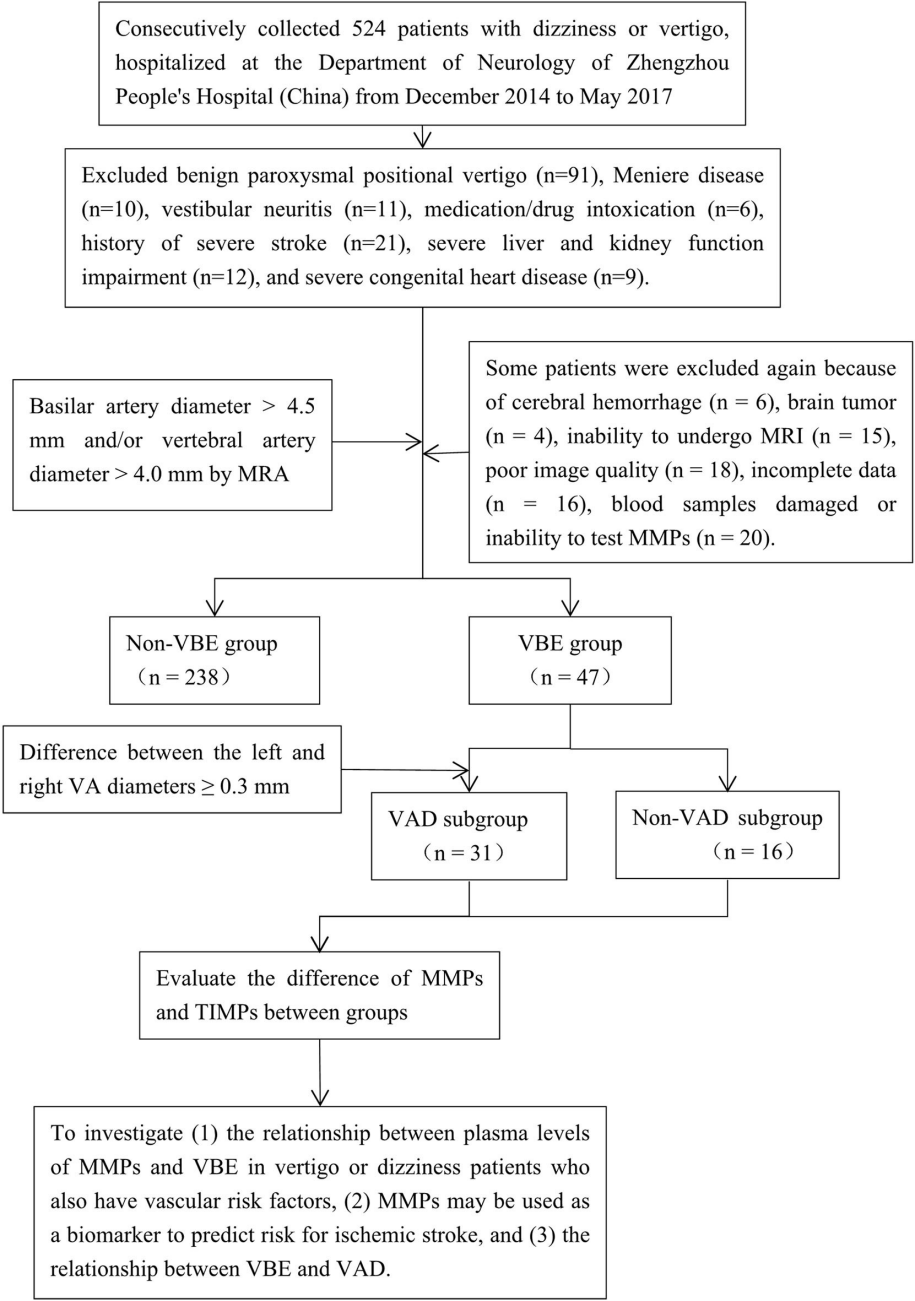
Flow-chart illustrating the selection process for this study of the association among MMP-9, vertebrobasilar ectasia (VBE), and vertebral artery dominance (VAD) in patients with vertigo or dizziness who also have vascular risk factors. MRA was used to identify patients with VBE and VAD among a group of 285 consecutive patients. Blood samples were drawn from patients that met the inclusion and exclusion criteria. The patients were divided into VBE-negative and VBE-positive groups, each of which was subdivided into non-VAD and VAD subgroups. Finally, the associations of MMPs, VBE, and VAD were analyzed.

We prospectively collected information about 524 consecutive patients with dizziness or vertigo hospitalized at the Department of Neurology of Zhengzhou People's Hospital from December 2014 to May 2017 and set up a cohort study. Notably, VBE patients who have vascular risk factors contributing to posterior circulation events usually present with dizziness or vertigo. A trained neurologist (D.P.Z) performed a neurological and vestibular examination on each patient, according to a standard protocol. Most patients also underwent confirmatory caloric testing of vestibular function. Inclusion criteria were as follows: age over 18 years; dizziness, vertigo or disequilibrium, spinning, or imbalance (with focal neurological or isolated symptoms); at least one vascular risk factor (arterial hypertension, diabetes mellitus, hyperlipidemia, hyperhomocysteinemia, history of stroke, drinking, smoking or coronary artery disease); and negative results on the Dixhall-pike and Roll tests. Exclusion criteria were dizziness or vertigo with benign paroxysmal positional vertigo (*n* = 91), Meniere disease (*n* = 10), vestibular neuritis (*n* = 11), medication/drug intoxication (*n* = 6), history of severe stroke (*n* = 21), severe liver and kidney function impairment (*n* = 12), and severe congenital heart disease (*n* = 9). All patients were evaluated by computed tomography (CT) and advised to undergo magnetic resonance imaging (MRI), generally after bedside evaluation and the drawing of blood samples. Patients were also asked to provide blood samples for measurement of MMP levels after admission. Then some patients were excluded again because of cerebral hemorrhage (*n* = 6), brain tumor (*n* = 4), inability to undergo MRI (*n* = 15), poor image quality (*n* = 18), incomplete data (*n* = 16), damage to the blood samples obtained, or inability to test MMPs (*n* = 20). Ultimately, 285 of the 524 patients with complete data were selected as research subjects (Figure 1). The reference standard for a stroke diagnosis was confirmation of acute stroke by neuroimaging, generally MRI with diffusion-weighted imaging (DWI) on the day of the index visit. Magnetic resonance angiography (MRA) was used to identify ectatic blood vessels. The basilar artery (BA) was considered ectatic if the diameter of the artery at the level of the pons was >4.5 mm (1). The vertebral artery (VA) was considered ectatic if the diameter of the V4 portion was >4.0 mm (2). Using these criteria, patients were classified as VBE-positive (18 with ischemic stroke, including 14 cases of posterior circulation ischemic stroke, and 29 cases without ischemic stroke) or VBE-negative (64 with ischemic stroke, including 9 cases of posterior circulation ischemic stroke, and 174 cases without ischemic stroke).

All participants signed an informed consent form before enrollment in the study. The ethics committee of Zhengzhou People's Hospital approved the research protocol.

### Ultrasonography

All patients were evaluated by stroke neurologists experienced in the use of cerebrovascular ultrasound using a high-resolution color-coded duplex sonography scanner (Philips iU22). Cervical vessels were examined with a high-frequency (5–10 MHz) linear probe. Carotid plaque and stenoses were graded according to validated criteria (14).

### MRI Analysis

MRI and MRA were performed using a 3.0-T scanner (GE Medical, Piscataway, NJ, USA), within 7 days of hospitalization. Conventional T2-weighted imaging, fluid-attenuated inversion recovery and diffusion-weighted imaging were obtained in the axial plane with sections of 5 mm thickness and 1-mm length. Three-dimensional time-of-flight (TOF) MRA was performed with a repetition time of 24 ms, echo time of 6 ms, FOV of 24 × 24 cm, and section thickness of 0.8–1.6 mm. Scanning results were obtained by reconstructing the image with maximum intensity projection. VA diameter was measured at three consecutive points from the bilateral VA junction (3 mm apart); only the maximum value was considered. BA diameter was measured at the mid-pons level on TOF source images.

**Figure 2:**
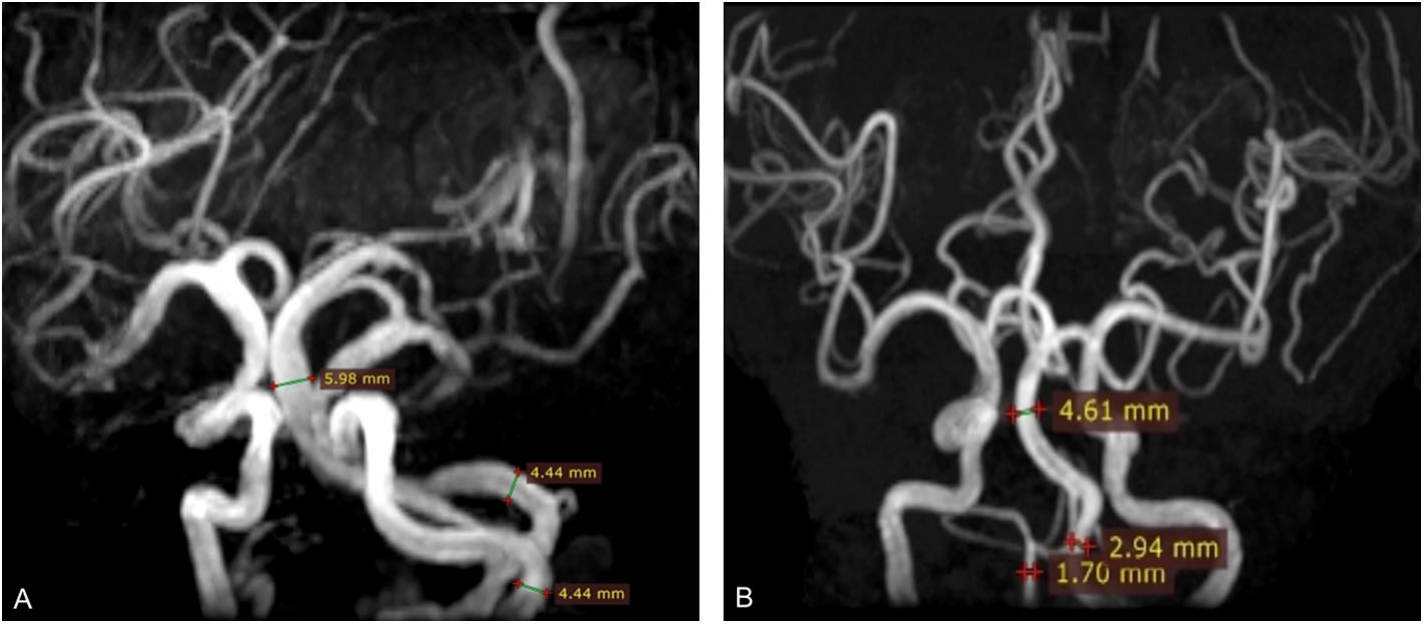
Three-dimensional time-of-flight (TOF) MRA shows basilar artery (BA) diameter was >4.5 mm (arrow). **(A)** BA diameter was 5.98 mm; vertebral artery (VA) diameter was 4.4 mm. **(B)** BA diameter was 4.61 mm; right vertebral artery (VA) diameter was 1.7 mm; left VA diameter was 2.94 mm. The difference in bilateral VA diameter was 1.24 mm.

MRI analysis was performed by two experienced neuroradiologists (S.Y. and M.Y.W.), who were blinded to clinical and demographic data. In the case of disagreement between 2 experts, a final decision was made based on consensus. In the present study, VBE was defined as BA diameter >4.5 mm, or VA diameter >4.0 mm. Vertebral artery dominance (VAD) was defined as difference between the left and right VA diameters ≥ 0.3 mm (19). Based on BA diameter and maximum bilateral VA diameter, study subjects were divided into a VBE-positive group and a VBE-negative group. VBE-positive subjects were divided in two subgroups, based on bilateral VA diameter data: a non-VAD subgroup (Figure 2A) and a VAD subgroup (Figure 2B).

### Serology

Venous blood samples were drawn within 1 day of admission, then clotted for 30 min at room temperature in serum separator tubes (SST) before centrifugation for 15 min at 1,000 × g. Plasma samples were frozen and stored at ≤-80°C until further use.

MMPs concentrations were determined by commercially available quantikine enzyme-linked immunosorbent assay (ELISA) (Human Total MMP-3 Quantikine ELISA Kit (SMP300), Total MMP-2 Quantikine ELISA Kit (SMMP200), Human MMP-9 Quantikine ELISA Kit (SMP900), Human TIMP-1 Quantikine ELISA Kit (STM100), Human TIMP-2 Quantikine ELISA Kit (DTM200), R&D Systems, Minneapolis, MN, USA). All samples were brought to room temperature before use, then analyzed in duplicate. Samples were diluted to the appropriate concentration. Samples or standard were added, then incubated for 2 h at room temperature on a horizontal orbital microplate shaker (0.12″ orbit) set at 500 ± 50 rpm, and then incubated for 2 h with conjugates: total MMP-2 conjugate, human MMP-3 conjugate, human MMP-9 conjugate, human TIMP-1 conjugate, or human TIMP-2 conjugate. Substrate solution was added, and samples were incubated for 30 min, until stop solution was added. Absorbance at 450 nm was measured with a microplate reader (Multiskan FC, Thermo) within 30 min. All results are expressed as nanogram per milliliter (n*g*/ml).

Cathepsin L concentration was detected by ancillary reagent ELISA kit (DUOsET ELISA Ancillary Reagent Kit 2 (DY008), R&D Systems, Minneapolis, MN, USA). Samples were preprocessed before beginning the assay protocol. Diluted capture antibody was coated on the microplate and incubated overnight. Binding was later blocked by incubating for a minimum of 1 h with reagent diluent. After excess antibody was washed away, plates were ready to receive samples. Samples or standards were added and incubated for 2 h. The detection antibody and streptavidin-horseradish peroxidase were added in sequence; samples were then incubated for 20 min. After adding substrate solution and 20 min of incubation, stop solution was added, followed by gentle tapping to ensure thorough mixing. A blue color developed in proportion to the amount of analyte present in the sample. The reaction was stopped when the color of the solution had turned to yellow. We determined the absorbance of the color immediately, using a microplate reader (Multiskan FC, Thermo) set to 450 nm. Results are expressed as pictogram per milliliter (pg/ml).

### Statistical Analysis

All statistical analyses were performed on the group of 285 selected patients. Values are expressed as mean ± SD or median ± quartile range. Baseline characteristics of patients with or without ischemic stroke were evaluated by univariate analysis with Student's *t*-test for continuous factors and the χ2 test for dichotomous factors. Fisher's exact test was used when expected cell frequency was <5. Potential risk factors (*P* < 0.20) were taken into the multivariate analysis with adjustment for age and gender. Levels of MMPs were compared between groups with the Mann-Whitney U-test. A receiver operating characteristic (ROC) curve was constructed to evaluate the predictive effects of MMPs on the occurrence of ischemic stroke in VBE patients and in patients with risk of posterior circulation ischemic events. Statistical testing was performed with a 2-tailed α level of 0.05. Data were analyzed with IBM SPSS version 20.0.

## Results

### Patient Characteristics

Of a total of 285 patients included in the study, 147 were male; 138 were female. Age ranged from 31 to 90 years (mean, 61.1±12.4 years). VBE was reported in 47 of the 285 patients with vertigo and at least one vascular risk factor (16.5%). Of the 82 patients with ischemic stroke, 34 strokes involved the pons; 12 involved the cerebellum; 2 involved the medulla oblongata; 6 involved the thalamus; 14 involved cortex or subcortex; 14 were multiple cerebral infarctions (simultaneously involving at least two structures mentioned above). Of the 82 ischemic stroke patients, 42 had focal neurological symptoms, and 40 had isolated vertigo or dizziness. Initial imaging occurred within 6 h of study examination in most cases (71%). Among the 74 patients for whom the time of symptom onset was known, imaging occurred within 72 h of symptom onset in 95% of cases; 3 patients were imaged at 4 days after onset, and one patient was imaged at 7 days after onset. Four patients with initial negative results on MRI underwent repeat MRI for unexplained signs suggesting brainstem localization (the time of the initial or follow-up examination).

Baseline characteristics and mean MMP levels of patients with vs, without ischemic stroke are shown in Table 1. Furthermore, we compared baseline characteristics of patients with VBE to those of patients without VBE (Table 2). There was a significant difference at risk of posterior circulation ischemic stroke between the VBE-positive group the VBE-negative group (*P* = 0.002). Univariate analysis showed that diabetes (*P* = 0.185) and hyperhomocysteinemia (*P* = 0.160) tended to increase risk for VBE (*P* < 0.20), but multivariate analysis with adjustment for age and gender showed that neither diabetes nor hyperhomocysteinemia increased risk for VBE (*P* > 0.05). There were no significant differences between the VBE-positive and VBE-negative groups in terms of age, male gender, hypertension, diabetes, dyslipidemia, history of vertigo, or carotid arteries plaques.

**Table 1:** Baseline characteristics of patients with vertigo or dizziness with increased vascular risk.

	**IS (*n* = 82)**	**Non-IS (*n* = 203)**	**Unadjusted**,	**Adjusted age and gender**,
			***P*-value**	***P-*value**
**Demographic characteristics**				
Age, y	61.3 ± 11.7	61.0 ± 12.6	0.858	0.830^†^
Male sex (*n*, %)	54 (65.9)	93 (45.8)	0.002	0.002^†^
Hypertension (*n*, %)	67 (81.7)	121 (59.6)	0.000	0.000^†^
Diabetes (*n*, %)	40 (48.8)	63 (31.0)	0.005	0.005^†^
Dyslipidemia (*n*, %)	51 (62.2)	123 (60.6)	0.802	
CAD (*n*, %)	18 (21.9)	47 (23.2)	0.827	
Hyperhomocysteinemia (*n*, %)	33 (40.2)	58 (28.6)	0.063	0.063^†^
Hyperuricemia (*n*, %)	3 (3.7)	11 (5.4)	0.764	
Smoking (*n*, %)	34 (41.5)	41 (20.2)	0.000	0.000^†^
Alcoholism (*n*, %)	20 (24.4)	26 (12.8)	0.016	0.016^†^
**Medical history**				
Vertigo (*n*, %)	1 (1.2)	7 (3.5)	0.446	
Strokes (*n*, %)	26 (31.7)	25 (12.3)	0.000	0.000^†^
**Ultrasound**				
Carotid stenosis (>50%) (*n*, %)	43 (52.4)	63 (31.0)	0.001	0.001^†^
Carotid plaques (*n*, %)	52 (63.4)	119 (58.6)	0.455	
**MRA**				
Intracranial arterial stenosis (>50%) (*n*, %)	20 (24.4)	15 (7.4)	0.000	0.000^†^
VBE	18 (21.9)	29 (14.3)	0.012	0.002^†^
**Serological test**				
MMP-2	234.87 (201.11, 288.24)	253.27 (222.50, 291.51)		0.038^‡^
MMP-3	12.24 (6.11, 18.33)	10.97 (6.93, 17.78)		0.729^‡^
MMP-9	495.13 (335.64, 799.48)	439.38 (270.97, 689.38)		0.030^‡^
TIMP-1	181.88 (165.22, 212.17)	177.02 (158.19, 203.14)		0.093^‡^
TIMP-2	85.70 (72.64, 94.13)	88.47 (79.01, 99.35)		0.074^‡^
Cathepsin L	2146.27 (1675.56, 2906.84)	2168.02 (1665.90, 2974.07)		0.967^‡^

*IS, ischemic stroke; CAD, coronary artery disease; MRA, magnetic resonance angiography; VBE, vertebrobasilar ectasia; MMP, matrix metalloproteinase; TIMP, tissue inhibitor of metalloproteinase*.

*Age is expressed as mean ± SD. MMP-2,-3,-9,TIMP-1,-2, and cathepsin L are expressed as median and percentile*.

† *P-values were calculated with multivariate analysis for continuous factors, including adjustment for age and gender, and the χ2 test for dichotomous factors*.

‡ *P-values were calculated with the Mann-Whitney U-test*.

**Table 2:** Characteristics of patients with vertigo or dizziness with vertebrobasilar ectasia.

	**VBE (*n* = 47)**	**Non-VBE (*n* = 238)**	**Unadjusted, *P*-value**	**Adjusted age and gender, *P-*value**
Age, y mean ± SD	61.7 ± 11.3	61.0 ± 12.6	0.734	
Male sex (*n*, %)	26 (55.3)	121 (50.8)	0.574	
Hypertension (*n*, %)	31 (65.9)	157 (65.9)	0.999	
Diabetes (*n*, %)	13 (27.7)	90 (37.8)	0.185	0.143
Dyslipidemia (*n*, %)	31 (65.9)	143 (60.1)	0.450	
CAD (*n*, %)	13 (27.7)	52 (21.9)	0.386	
Hyperhomocysteinemia (*n*, %)	11 (23.4)	80 (33.6)	0.160	0.095
Hyperuricemia (*n*, %)	3 (6.4)	11 (4.6)	0.709	
**Medical history**				
Vertigo (*n*, %)	2 (4.3)	6 (2.5)	0.623	
Strokes (*n*, %)	6 (12.8)	45 (18.9)	0.315	
Smoking (*n*, %)	12 (25.5)	63 (26.5)	0.894	
Alcoholism (*n*, %)	8 (17.0)	38 (16.0)	0.857	
Carotid stenosis (>50%) (*n*, %)	18 (38.3)	88 (37.0)	0.864	
Intracranial arterial stenosis (>50%) (*n*, %)	4 (8.5)	31 (13.0)	0.389	
Carotid plaques (*n*, %)	28 (59.6)	143 (60.1)	0.948	
Acute posterior circulation ischemic stroke (*n*, %)	14 (29.8)	9 (3.8)	0.002	0.002

*CAD, coronary artery disease; VBE, vertebrobasilar ectasia*.

*P-values were calculated with multivariate analysis for continuous factors and the χ2 test for dichotomous factors*.

### Associations of MMP-2, MMP-3, MMP-9, TIMP-1, TIMP-2, and Vertebrobasilar Ectasia (VBE)

As shown in Table 3, mean MMP-9 levels were higher in the VBE-positive group than in the VBE-negative group (*P* = 0.022). Mean levels of MMP-2 tended to be higher in the VBE-negative group than in the VBE-negative group (*P* = 0.054). Mean levels of MMP-3 were higher in the VBE-negative group than in the VBE-positive group, but the difference was not significant (*P* = 0.611). Conversely, mean levels of TIMP-1,−2 were higher in the VBE-negative group than in the VBE-positive group, but this trend did not achieve statistical significance (*P* = 0.229, *P* = 0.588, respectively). Mean levels of cathepsin L were higher in the VBE-negative group, compared with the VBE-positive group, but the difference was not statistically significant (*P* = 0.060).

**Table 3:** Association between VBE and plasma level of metalloproteinases.

	**VBE+ (*n* = 47)**	**VBE– (*n* = 238)**	***P-*value**
MMP-2^*^	238.78 (210.91, 263.49)	250.65 (217.07, 295.74)	0.054
MMP-3^*^	10.67 (6.70, 14.24)	11.29 (6.63, 18.17)	0.611
MMP-9^*^	571.09 (395.90, 748.40)	442.59 (287.92, 698.36)	0.022
TIMP-1^*^	174.33 (156.03, 197.07)	181.16 (160.54, 204.65)	0.229
TIMP-2^*^	86.17 (77.34, 94.65)	86.37 (77.37, 98.68)	0.588
Cathepsin L^*^	1953.47 (1489.39, 2666.54)	2198.53 (1696.49, 3021.30)	0.060

*VBE, vertebrobasilar ectasia; MMP, matrix metalloproteinase; TIMP, tissue inhibitor of metalloproteinase*.

* *MMP-2,−3,−9, TIMP-1 and−2 are expressed as ng/ml; Cathepsin L is expressed as pg/ml. Values are expressed as median percentiles*.

*P-values calculated with the Mann-Whitney U-test*.

### MMPs and VAD in VBE-Positive Patients

As shown in Table 4, of the 47 patients with VBE, 31 patients were assigned to the VAD subgroup, and 16 patients were assigned to the non-VAD subgroup. Differences in levels of MMP-2,−3,−9, TIMP-1,−2, and Cathepsin L were detected between subgroups. Mean levels of MMP-2,−9, and TIMP-2 were higher in the VAD subgroup, but this difference was not significant (*P* = 0.836, *P* = 0.809, *P* = 0.370, respectively). Mean levels of MMP-3 were similar between subgroups (*P* = 0.301). In contrast, mean levels of TIMP-1 and cathepsin L were higher in the non-VAD subgroup, compared with the VAD subgroup, but this difference was not statistically significant (*P* = 0.084, *P* = 0.890, respectively).

**Table 4:** Association between MMP levels and vertebral artery dominance (VAD) in VBE-positive group.

	**VAD+ (*n* = 31)**	**VAD– (*n* = 16)**	***P-*value**
MMP-2^*^	241.42 (211.62, 268.78)	223.62 (192.58, 241.48)	0.836
MMP-3^*^	10.69 (6.69, 14.09)	10.46 (6.95, 19.61)	0.301
MMP-9^*^	586.95 (395.63, 59.94)	571.09 (377.03, 762.19)	0.809
TIMP-1^*^	170.79 (151.27, 188.61)	197.07 (177.23, 218.53)	0.084
TIMP-2^*^	86.37 (77.61, 94.77)	84.10 (73.33, 91.60)	0.370
Cathepsin L^*^	1976.13 (1475.51, 2567.73)	1793.77 (1382.03, 3279.65)	0.890

*MMP, matrix metalloproteinase; TIMP, tissue inhibitor of metalloproteinase*.

* *MMP-2,−3,−9, TIMP-1 and−2 are expressed as ng/ml; Cathepsin L is expressed in pg/ml. Values are expressed as median and percentiles. P-values were calculated with the Mann-Whitney U-test*.

### Predictive Effects of MMPs on the Occurrence of Ischemic Stroke

**Figure 3:**
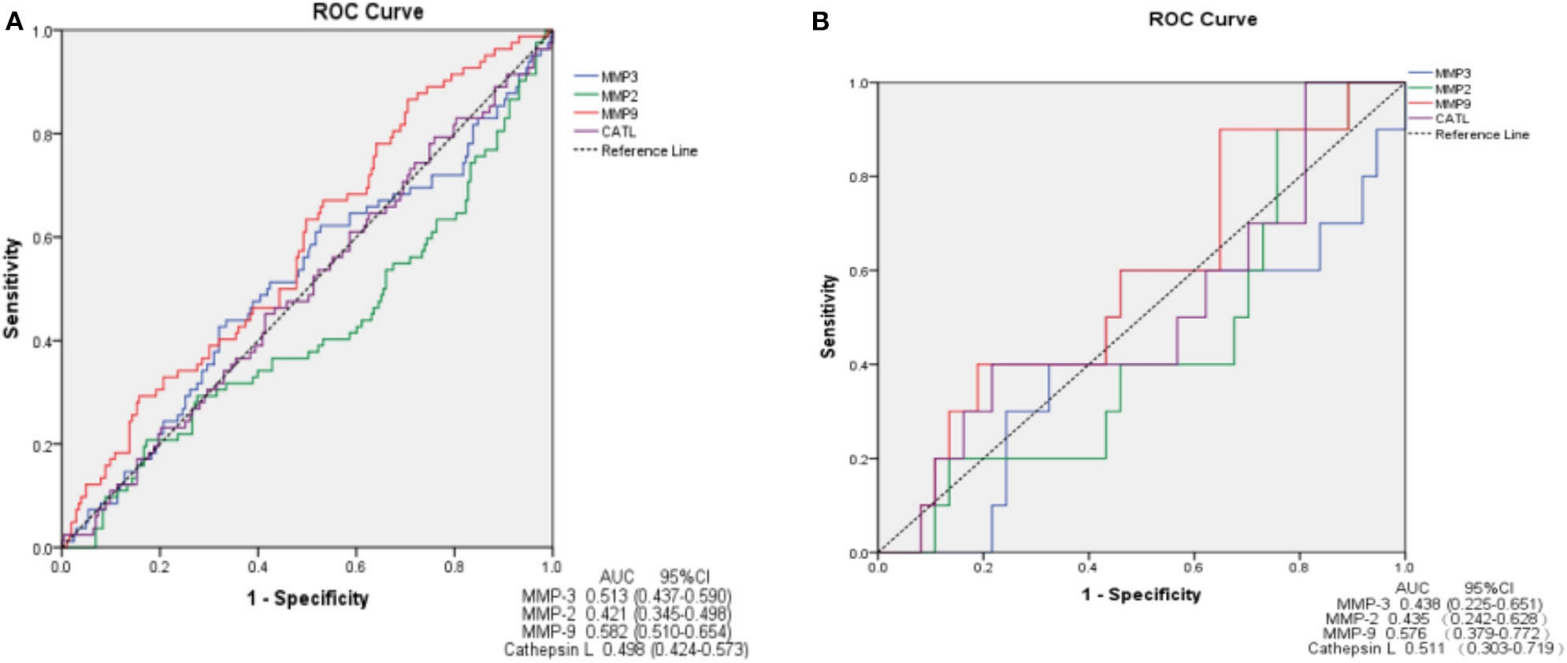
ROC curve demonstrating sensitivity as a function of 1-specificity for MMPs. MMP-9 shows greater sensitivity and specificity for prediction of ischemic stroke in patients presenting with vertigo or dizziness who also have vascular risk factors (AUC = 0.582) **(A)**. MMPs show no sensitivity or specificity for prediction of ischemic stroke in VBE patients **(B)**. AUC, area under the curve; CI, confidence interval.

In ROC analysis, MMP-9 had a significantly predictive effect on ischemic stroke (area under the curve, AUC = 0.582, 95%CI, 0.510–0.654, *P* = 0.030). MMP-2, MMP-3 and cathepsin L showed a smaller AUC and had no predictive effects on ischemic stroke (Figure 3A). We further determined the predictive effects of MMPs on ischemic stroke in 47 VBE patients. We found no significant predictive effect of any MMP on ischemic stroke in these patients (Figure 3B).

## Discussion

The present study demonstrated that high plasma levels of MMP-9 are associated with VBE, independent of the presence of VAD, and may predict the occurrence of ischemic stroke in patients with vertigo or dizziness with vascular risk factors [increased risk for posterior circulation ischemic events (17)]. Should our findings be further confirmed, MMP-9 could serve as a useful biomarker for identifying infarction in patients with vertigo or dizziness. MMP inhibitors could be used to treat patients with VBE.

The prevalence of VBE observed in the present study of individuals at higher risk for posterior circulation ischemic events was 16.5%. This value is close to the figure of 15.5% reported in the Northern Manhattan Study, which investigated the frequency of VBE in an unselected population and assessed various diagnostic methods (20), suggesting the lack of selection bias for VBE in the present study population. Previous studies (3) that showed that age, male gender, hypertension, diabetes, and current smoking are risk factors for VBE did not find any association between VBE and either recurrent stroke or hemorrhage. However, on the contrary, we found that vascular risk factors did not increase risk for VBE, and that VBE may be associated with acute stroke. Notably, a prospective, observational study on 86 patients with acute transient vestibular syndrome demonstrated that the frequency of vascular risk factors was similar between patients with stroke and patients with other disorders (21). This discrepancy may be attributed to differences in study population. Compared with previous studies (22), our study included a higher proportion of patients without stroke. Levels of MMPs, especially MMP-9, are known to be influenced by several general conditions such as hypertension, smoking, and diabetes. In the present study, it was the balance of vascular risk factors between the VBE-positive and VBE-negative groups that confirmed the lack of an impact of comorbidities on plasma MMP-9 levels. This finding supports our initial hypothesis that high levels of MMP-9 are associated VBE.

MMPs are a family of zinc-binding proteolytic enzymes that can degrade extracellular matrix proteins, including elastinase, collagen, and glycoprotein polymers. This process may thin or destroy the internal elastic lamina of arteries. Lamblin et al. (23) examined polymorphisms in genes for MMPs in patients with coronary artery disease (CAD) with coronary aneurysms, compared with patients with CAD but without coronary aneurysms. The results showed that the MMP-3 5A allele was associated with the occurrence of coronary aneurysm. Similar findings were reported for abdominal aortic aneurysm (24, 25). Furthermore, tetracycline therapy may decrease the risk for hemorrhage in patients with brain vascular malformations such as arteriovenous malformation and intracranial aneurysm (26). Interestingly, a recent study that investigated MMPs and brain arterial remodeling confirmed that high MMP-9 expression, alone or combined with low TIMP-2 expression, was associated with dolichoectasia in HIV-negative individuals; high expression of MMP-9, accompanied by expression of caspase 3, was associated with dolichoectasia in HIV-positive individuals (10). MMP-2 levels were found to be associated with cervical/intracranial carotid artery dolichoarteriopathies (11). Experiments in a mouse model of elastase-induced VBE demonstrated that arterial wall dilation was characterized by disruption of the internal elastic lamina, the infiltration of inflammatory cells, and high media expression of MMP-2 and MMP-9 (12). In the current study, we found that high plasma MMP-9 levels were associated with VBE and predicted ischemic stroke in patients with vertigo or dizziness. In combination with previous results from our group, these findings suggest that the pathological mechanism of VBE involves MMP-9. However, the usefulness of MMP-9 in predicting ischemic stroke should be studied further. Our findings may thus provide preliminary evidence that MMPs represent a target for intervention in this patient population.

Hemodynamic-induced vascular remodeling has previously been observed. VBD is associated with VAD (27), and imbalanced flow through the vertebral artery is an important hemodynamic contributor to VBD (14). One group of researchers used the flow-augmented common carotid artery model in mice to show that macrophages infiltrated the adventitia of the flow-augmented common carotid artery (28). Furthermore, high levels of MMP-9 expression were observed in the dilated artery wall, and flow-induced outward vascular remodeling was significantly reduced in mice treated with an MMP-9 inhibitor, as well as in MMP-9 knockout mice (29). Aoki et al. (30) found that macrophages infiltrated the arterial wall of experimentally induced rat cerebral aneurysms and expressed MMP-2 and MMP-9; in addition, treatment with an MMP inhibitor reduced the proportion of advanced aneurysms in the rat model. The current study suggested that increased MMP levels are associated with VBE but caused by vascular remodeling induced by hemodynamic changes associated with VAD. However, in order to provide mechanistic evidence, genetic or pharmacological tools should be used to develop an animal model that can provide hard evidence of the causality relationship between MMPs and VBE.

This study had several limitations. First, this prospective, cross-sectional study was performed in a single center; the limited data set may have resulted in selection bias. Second, we focused on VBE patients with dizziness or vertigo, but asymptomatic VBE patients were ineligible for inclusion, which may have contributed to selection bias. Third, the analyses performed failed to distinguish between BA vs. VA ectasia. Nonetheless, this was the largest study to date to analyze the prevalence and frequency of VBE and associated biochemical etiology in patients with vertigo or dizziness and increased vascular risk. In order to thoroughly elucidate the relationships between MMP-9 and VBE, future studies will need to increase the size of the sample population and conduct regular patient follow-up.

In conclusion, the present findings suggest that high plasma levels of MMP-9 are associated with VBE and independent of VAD, and that MMP-9 predicts the occurrence of ischemic stroke in patients at higher risk of posterior circulation ischemic events. Future research should explore the association between VBE and levels of MMPs, which may predict ischemic stroke, especially among those at high risk for posterior circulation ischemic stroke. MMP inhibition may become the treatment of choice for VBE.

## Data Availability Statement

The raw data supporting the conclusions of this article will be made available by the authors, without undue reservation, to any qualified researcher.

## Ethics Statement

The ethics committee of Zhengzhou People's Hospital approved the research protocol. The patients/participants provided their written informed consent to participate in this study. Written informed consent was obtained from the individual(s), and minor(s)' legal guardian/next of kin, for the publication of any potentially identifiable images or data included in this article.

## Author Contributions

DZ and MZ conceived this study and provided financial support. YP and DL analyzed the whole data and wrote the draft manuscript. HZ and FL collected the clinical data of the patients. SY and JL collected and analyzed the image data. TW tested the serum levels of MMPs and TIMPs. All authors contributed to the article and approved the submitted version.

## Conflict of Interest

The authors declare that the research was conducted in the absence of any commercial or financial relationships that could be construed as a potential conflict of interest. The reviewer NL and handling editor declared their shared affiliation at the time of review.
